# The role of neighborhood socioeconomic environment in the association between glycemic control and the developing brain

**DOI:** 10.1007/s11682-026-01211-0

**Published:** 2026-10-14

**Authors:** Anisha Chandra, Eustace Hsu, Shan Luo

**Affiliations:** 1 Keck School of Medicine, University of Southern California, Los Angeles, CA USA; 2 Division of Endocrinology and Diabetes, Keck School of Medicine, USC, Los Angeles, CA USA; 3 Department of Psychology, USC, Los Angeles, CA USA; 4 Neuroscience Graduate Program, USC, Los Angeles, CA USA; 5 Diabetes and Obesity Research Institute, USC, 2250 Alcazar Street, CSC 215, Los Angeles, CA 90089 USA

**Keywords:** Glycemic control, Brain structure, Neighborhood socioeconomic deprivation, Adolescent development

## Abstract

Metabolic health and socioeconomic environments may jointly shape neurodevelopment during adolescence, a sensitive period of brain maturation. This cross-sectional study investigated overall and neighborhood socioeconomic deprivation moderated associations between glycemic control and brain structure in youth. Data were analyzed from 705 healthy 10–13-year-olds participating in the Adolescent Brain and Cognitive Development (ABCD) Study® across 21 sites in the United States. Glycemic control was assessed using hemoglobin A1c (HbA1c), brain structure was evaluated via magnetic resonance imaging (MRI), and neighborhood deprivation was measured with the Area Deprivation Index (ADI). Mixed-effects models examined associations between HbA1c and brain structural measures while controlling for sociodemographic covariates, with additional analyses stratified by ADI tertiles. Higher HbA1c was associated with lower mean cortical thickness and smaller total cortical gray matter volume. Each one percent increase in HbA1c corresponded to a 0.024 mm reduction in mean cortical thickness and a 9,611 mm^3^ reduction in total cortical gray matter volume. Regionally, higher HbA1c was associated with thinner cortex and smaller gray matter volumes primarily within frontal, cingulate and occipital areas. Exploratory analysis found that higher HbA1c was associated with lower total cortical gray matter volume among youth living in medium and high ADI neighborhoods, but not low ADI neighborhoods, whereas higher HbA1c was associated with lower mean cortical thickness only among youth living in high ADI neighborhoods. These findings highlight the interplay between metabolic health and neighborhood deprivation, supporting a model in which socioeconomic disadvantage may magnify the neurodevelopmental impact of metabolic variation, even within the non-diabetic range.

**Supplementary Information:**

The online version contains supplementary material available at https://doi.org/10.1007/s11682-026-01211-0.

## Introduction

Obesity and type 2 diabetes (T2D) rates have continued to rise over the past several decades. Approximately one in five children in the US are living with obesity, predisposing them to diabetes (Centers for Disease Control & Prevention, 2026). The estimated prevalence of T2D in youth nearly doubled between 2001 and 2017, with higher incidence and T2D related complications and comorbidities in youth from lower socioeconomic backgrounds (Lawrence et al., 2021). Compared to previous generations, the current generation of youth is more overweight and T2D is being diagnosed at earlier ages, affirming that these conditions are key public health priorities (Rodriquez & O’Sullivan, 2023). T2D in children comes with several potential complications, including neurological issues.

The developing brain is highly reliant on glucose for fuel and growth. Functional imaging studies have shown that acute changes in circulating glucose alter activity in hypothalamic and corticolimbic circuits involved in energy homeostasis and food motivation (Page et al., 2011; Smeets et al., 2005). It is less known how *chronic* exposure to high levels of glucose impacts the developing brain. Structural imaging studies have found that youth with T2D had smaller global and regional gray matter volumes, primarily in the prefrontal and limbic regions (e.g., striatum, hippocampus, amygdala, thalamus) (Bruehl et al., 2011; Nouwen et al., 2017; Redel et al., 2018; Rofey et al., 2015). Such structural abnormalities may manifest as impaired cognitive function, a finding observed in youth with T2D (Redel et al., 2018; Yau et al., 2010). Moreover, higher hemoglobin A1c (HbA1c) was associated with smaller prefrontal volumes and greater cerebral atrophy in youth with T2D, suggesting that poor glycemic control may contribute to these observed structural brain abnormalities in youth with T2D (Bruehl et al., 2011).

While prior studies have demonstrated brain structure impairments in youth with T2D, it remains unknown whether the changes in the brain occur before onset of diabetes or consequences of diabetes. Furthermore, existing studies have not shown how individual variabilities in HbA1c relate to brain structure in healthy controls, where early alterations may indicate vulnerability before diabetes develops. Thus, it remains unknown the impact of glucose control on brain structure in typically developing youth prior to onset of T2D.

Early brain changes linked to glucose regulation may not occur in isolation but within a broader socioeconomic context. A growing body of evidence shows that higher neighborhood socioeconomic deprivation was associated with poorer glycemic control in youth with diabetes (Khanolkar et al., 2019; Queen et al., 2017; Tremblay et al., 2022). Neighborhood disadvantage has also been associated with smaller cortical surface area and gray matter volumes and lower regional cortical thickness in youth (Hackman et al., 2021; Rakesh et al., 2022). While several studies have highlighted racial-ethnic disparities underlying T2D in youth, it remains unknown whether different neighborhoods have differential relationships between HbA1c and the brain.

The purpose of our study was to evaluate the relationship between glycemic control and youth brain structures, specifically how neighborhood socioeconomic environment moderates these relationships. We hypothesized that higher HbA1c would be associated with lower structural brain measures, particularly in the prefrontal and limbic regions, and that neighborhood socioeconomic deprivation would amplify these associations.

## Methods

### Study design

The ABCD® Study is a longitudinal study of adolescent development. Participants aged 9–10 years old were initially recruited from 21 study sites across the U.S. between September 2016 and August 2018. Details for the ABCD Study have been reported previously (Garavan et al., 2018). For the current analysis, data were obtained from the ABCD® 4.0 data release (Adolescent Brain Cognitive Development Study Consortium, 2021), and focused on two-year follow-up between 3/3/2019 and 3/17/2020, when imaging and HbA1c data were collected. Our final sample (N = 705) consisted of participants with quality-controlled neuroimaging measurements and complete data for all outcomes of interest, and covariate variables. Participants with missing data for any analysis variable were excluded. A flowchart summarizing the sample selection and inclusion/exclusion criteria is provided in Supplemental Fig. S1and a table of characteristics of included and excluded participants is provided in Supplemental Table S1.

### HbA1c

Venous blood samples were collected into EDTA (purple-top) tubes and shipped, either ambient or frozen, to a processing lab for analysis. HbA1c (%) was measured in whole blood as a measure of long-term glycemic control, reflecting average blood glucose levels over the prior 3 months (Arslanian et al., 2018). Blood collection was suspended in March 2020 because of the COVID-19 pandemic; therefore, HbA1c data in Release 4.0 were available only for 2-year follow-up visits completed before that date. Participants were excluded with a parental-reported diabetes diagnosis at any time-point in the study, or with HbA1c of at least 6.5 (*n* = 9).

### Neuroimaging

MRI data collections were optimized and harmonized for 3-Tesla scanners across multiple platforms (Casey et al., 2018; Hagler et al., 2019). Cortical surface reconstruction and subcortical segmentation was completed via FreeSurfer (version 7.1.1), producing estimates of global and regional cortical thickness (mm), cortical surface area (mm^2^), and cortical and subcortical gray matter, and white matter volume (mm^3^).

Quality control (QC) was conducted to evaluate each cortical surface reconstruction. Images were excluded for failure to pass QC, as well as for abnormal radiological findings. Additionally, the top and bottom 1% of each neuroimaging measure was winsorized (via ‘winsor’) for outlier detection.

### Imaging data harmonization

Neuroimaging data was harmonized using ComBat, an empirical Bayes-based method for harmonizing data across scanners (Fortin, 2023; Fortin et al., 2018). Harmonization was processed (via ‘neuroCombat’) using the unique scanner identification as the “batch” effect, and sex, age, and HbA1c as biological covariates. In this way, the scanner-related variances between sites were removed while the biological variances were retained.

### Area deprivation index

Primary address was reported by the caregiver and matched with the 2010 US census tracts to identify neighborhoods. Area Deprivation Index (ADI) was calculated for individual census tracts based on 17 characteristics derived from the American Community Survey (2011–2015) (Singh, 2003). ADI is a comprehensive measurement of neighborhood socioeconomic deprivation, with higher ADI scores representing greater neighborhood deprivation (Kind et al., 2014). National percentiles of ADI were obtained by ranking across census tracts, to obtain an ADI percentile score corresponding to each participant’s residential history. ADI percentile was grouped into tertiles (via ‘split_quantile’; low: ≤ 25th percentile, medium: > 25th to 45th percentile, high: > 45th percentile) for ease of interpretation.

### Covariates

Sex was categorized as the child’s sex at birth. Age was the child’s age in months at the study visit. Race/ethnicity was categorized as Hispanic, non-Hispanic White, non-Hispanic Black, non-Hispanic Asian, and Other. Parental education was modeled as a binary variable indicating whether at least one parent has obtained a bachelor’s degree. Yearly household income was quantified in three categories: less than $50,000, between $50,000 and $99,999, and at least $100,000. Pubertal stage was calculated using the parent-reported pubertal development scale (Petersen et al., 1988).

### Data and software availability

Data used here were obtained from the ABCD® Study (https://abcdstudy.org), held in the National Institute of Mental Health (NIMH) Data Archive (NDA).

### Statistical analysis

Linear mixed effects models were conducted to examine associations between HbA1c (independent variable) and global brain measurements (dependent variables), adjusting for covariates. Global brain measurements included total cortical surface area, mean cortical thickness (CT), total cortical and subcortical gray matter volume (GMV), and cerebral white matter volume. Family ID was included as random effects to account for shared family membership; age, sex, race/ethnicity, parental education, household income, pubertal stage, and handedness were included as covariates. Intracranial volume (ICV) was included as an additional covariate in models of volumetric measures.

Region-of-interest (ROI) analyses were completed to follow up with significant associations of HbA1c with global brain measures. Cortical GMV and mean CT of 34 ROIs defined bilaterally on the Desikan-Killiany atlas were included. Correlations in each ROI were assessed using models predicting bilateral brain structure based on HbA1c by hemisphere interaction. Family ID was included as random effects, and subject identification was included as a random effect to pool across hemispheres. The same covariates for global measures were applied here.

To further study the impact of neighborhood socioeconomic deprivation on the relationships between HbA1c and brain, interaction of HbA1c x ADI was examined on brain measurements identified as significant in the whole cohort model, followed up by estimated HbA1c slopes within each tertile. Family ID was modeled as a random effect. Age, sex, race/ethnicity, pubertal stage, and handedness were included as covariates, with ICV as an added covariate for volumetric models. Parental education and household income were not included as covariates because they are closely related measures of socioeconomic status that capture constructs similar to neighborhood deprivation. Linear mixed effects models were fitted in R with the *lme4* package (via ‘lmer’) (Bates et al., 2015). Standardized betas (variables in the final dataset were transformed via ‘scale’) were reported, representing the standard-deviation increase in the dependent variable corresponding to a one standard-deviation increase in HbA1c. 95% Wald confidence intervals were calculated based on the local curvature of the likelihood surface. *P*-values were calculated using Satterthwaite’s method in the *lmerTest* package (Kuznetsova et al., 2017). Tests of significance (2-tailed) were corrected for multiple comparisons using the Benjamini–Hochberg false discovery rate (FDR) correction (via ‘p.adjust’), with *P* < 0.05 as the corrected threshold for significance. Relationships of regional brain measures with HbA1c (pooled across hemispheres), as well as estimated HbA1c slopes within each ADI tertile, were reported using the *emmeans* package (via ‘emtrends’) (Lenth 2026).

## Results

### Demographics

Sample characteristics are shown in Table 1. The average age of participants was 11.9 years with a range between 10 years 7 months, and 13 years 2 months. 43.3% of participants were female, 16.6% of participants were Hispanic or Latino, 60.7% non-Hispanic White, 10.4% non-Hispanic Black, 1.3% Asian, and 11.1% Other race. 21.7% of participants lived in households with less than $50,000/yr income, 28.1% with between $50,000/yr and $99,999/yr, and 50.2% above $100,000/yr. 65.0% of participants had at least one parent who graduated college. 22.8% of participants were pre-pubertal, 26.4% were in early puberty, 33.3% were in mid-puberty, 16.6% were in late puberty, and 0.9% were post-puberty. HbA1c was correlated with race/ethnicity (*F*_4,514_ = 9.68, *P* < 0.001) and parental education (*F*_1,567_ = 13.99, *P* < 0.001).

**Table 1:** Participant demographics

Characteristic	Mean/N	SD/%
Age (Years)	11.9	0.65
Sex		
Male	400	56.7%
Female	305	43.3%
Race/Ethnicity		
White	428	60.9%
Black or African American	73	10.2%
Hispanic or Latino	117	16.5%
Asian	9	1.3%
Other	78	11.1%
Family Income		
< $50,000	153	21.7%
≥ $50,000 and < $100,000	198	28.1%
≥ $100,000	354	50.2%
Parental Education (highest)		
No Bachelor’s Degree	247	35.0%
Bachelor’s Degree or Higher	458	65.0%
Pubertal Stage		
Pre-Puberty	161	22.8%
Early Puberty	186	26.4%
Mid-Puberty	235	33.3%
Late Puberty	117	16.6%
Post Puberty	6	0.9%
Area Deprivation Index (ADI)	39.5	24.8
HbA1c (%)	5.13	0.27

### HbA1c and global and regional brain measures

**Fig. 1:**
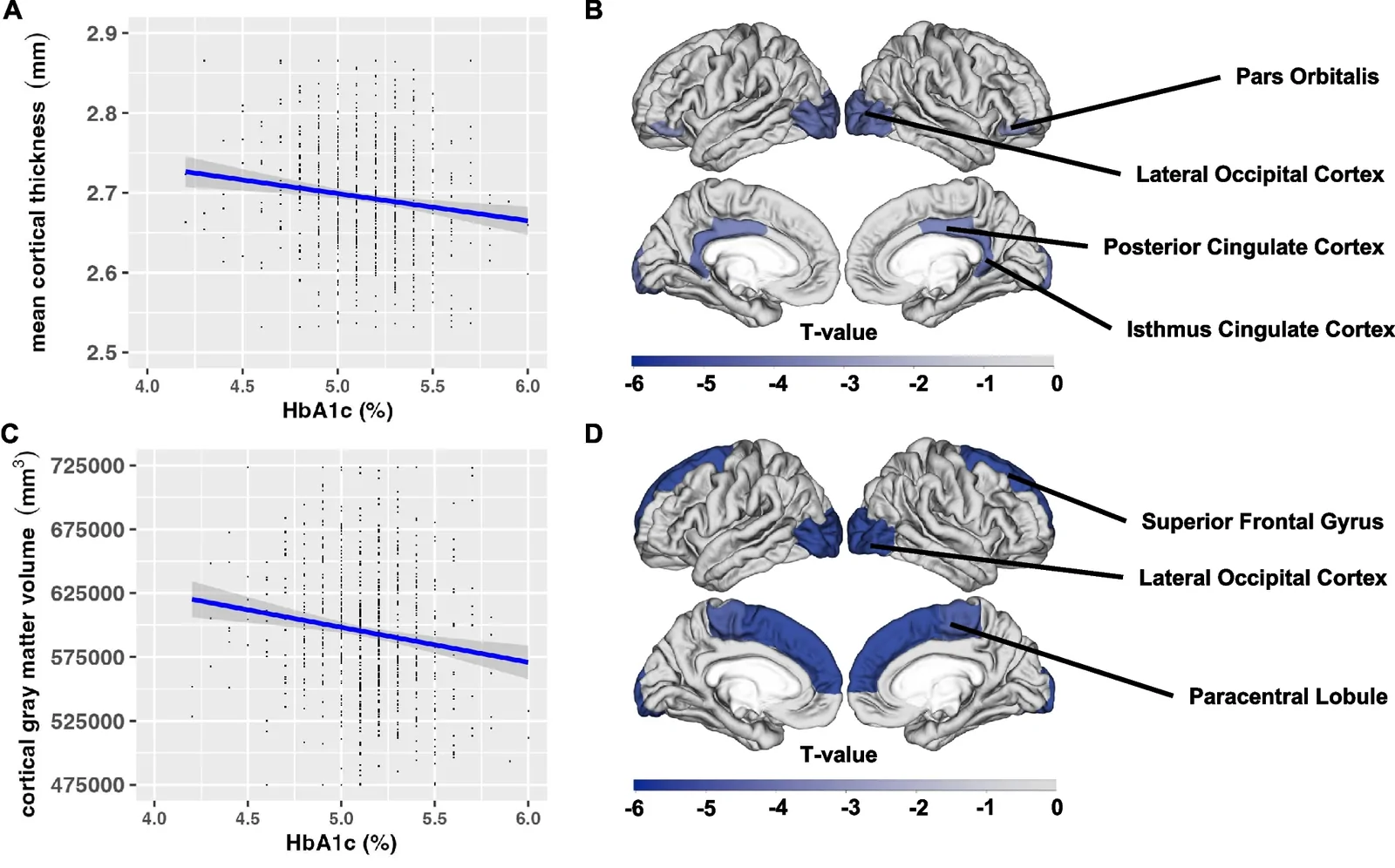
Association of hemoglobin A1c (HbA1c) levels (%) with global and regional brain measurements. **A** Scatterplot displays the relationship of HbA1c % with mean cortical thickness (CT). One percent increase in HbA1c was associated with a 24 mm or 0.9% decrease in mean CT. **B** T value denotes regions significantly associated with GDM exposure from linear mixed effects models (FDR corrected P < 0.05), where family ID, as well as subject, were included as random effects, and handedness, intracranial volume, age, sex, pubertal status, race/ethnicity, family income, and parental education were included as fixed effected. All regions of interest analysis were bilateral in the models. **C** Scatterplot displays the relationship of HbA1c % with total cortical gray matter volume (GMV). One percent increase in HbA1c was associated with a 9,611 mm^3^ or 1.6% decrease in total cortical GMV. **D** Associations between HbA1c and child regional cortical thickness in significant regions of interest. T value denotes regions significantly associated with GDM exposure from linear mixed effects models (FDR corrected *P* < 0.05), where family ID, as well as subject, were included as random effects, and handedness, age, sex, pubertal status, race/ethnicity, family income, and parental education were included as fixed effects. All regions of interest analysis were bilateral in the models

Higher HbA1c was associated with lower mean CT (β (95% CI) = −0.090 (−0.164, −0.015), FDR corrected* P* = 0.046) and smaller total cortical GMV (β (95% CI) = −0.048 (−0.085, −0.010), FDR corrected* P* = 0.046) (Fig. 1). One percent increase in HbA1c was associated with a −0.024 mm decrease in mean CT and 9,611 mm^3^ decrease in total GMV. HbA1c was not significantly associated with total cortical surface area, total subcortical GMV, and cerebral white matter volume (Table 2).

**Table 2:** Associations between HbA1c and global brain measurements

Global Brain Measurements	β (95% CI)*	FDR-adjusted *P*-value†
Total Cortical Surface Area	−0.009 (−0.069, 0.052)	0.782
Mean Cortical Thickness	−0.090 (−0.164, −0.015)	**0.046**
Cortical Gray Matter Volume	−0.048 (−0.085, −0.010)	**0.046**
Subcortical Gray Matter Volume	−0.028 (−0.069, 0.013)	0.306
Cerebral White Matter Volume	0.010 (−0.027, 0.047)	0.753

^*^ Standardized β (95% CI) from linear mixed effects models. Positive β indicates higher HbA1c is associated with larger brain measurement, negative β indicates the opposite relationship

^**†**^ Multiple comparisons are conducted with Benjamini–Hochberg FDR correction. Boldface indicates significance at the corrected threshold of *P* < 0.05

ROI analysis of GMV found that higher HbA1c was associated with smaller GMV in bilateral lateral occipital cortex (β (95% CI) = −0.093 (−0.148, −0.039), FDR corrected* P* = 0.025), paracentral lobule (β (95% CI) = −0.079 (−0.132, −0.026), FDR corrected* P* = 0.040), and superior frontal gyrus (β (95% CI) = −0.078 (−0.127, −0.030), FDR corrected* P* = 0.027) (Supplemental Table S2, Fig. 1). A one percent increase in HbA1c was associated with 1402 mm^3^ decreases in volume of the lateral occipital cortex, 374 mm^3^ in the paracentral lobule, and 1976 mm^3^ in the superior frontal cortex.

ROI analysis of CT found greater HbA1c was correlated with thinner isthmus cingulate cortex (β (95% CI) = −0.107 (−0.172 =, −0.043), FDR corrected* P* = 0.018), thinner lateral occipital cortex (β (95% CI) = −0.101 (−0.170, −0.033), FDR corrected* P* = 0.026), thinner pars orbitalis (β (95% CI) = −0.093 (−0.157, −0.029), FDR corrected* P* = 0.037), and posterior cingulate cortex (β (95% CI) = −0.107 (−0.171, −0.044), FDR corrected* P* = 0.018) (Supplemental Table S3, Fig. 1). A one percent increase in HbA1c was associated with 0.054 mm decreases in mean thickness of isthmus cingulate, 0.040 mm in lateral occipital cortex, 0.055 mm in pars orbitalis, and 0.043 in posterior cingulate cortex.

### HbA1c, ADI, and brain

**Fig. 2:**
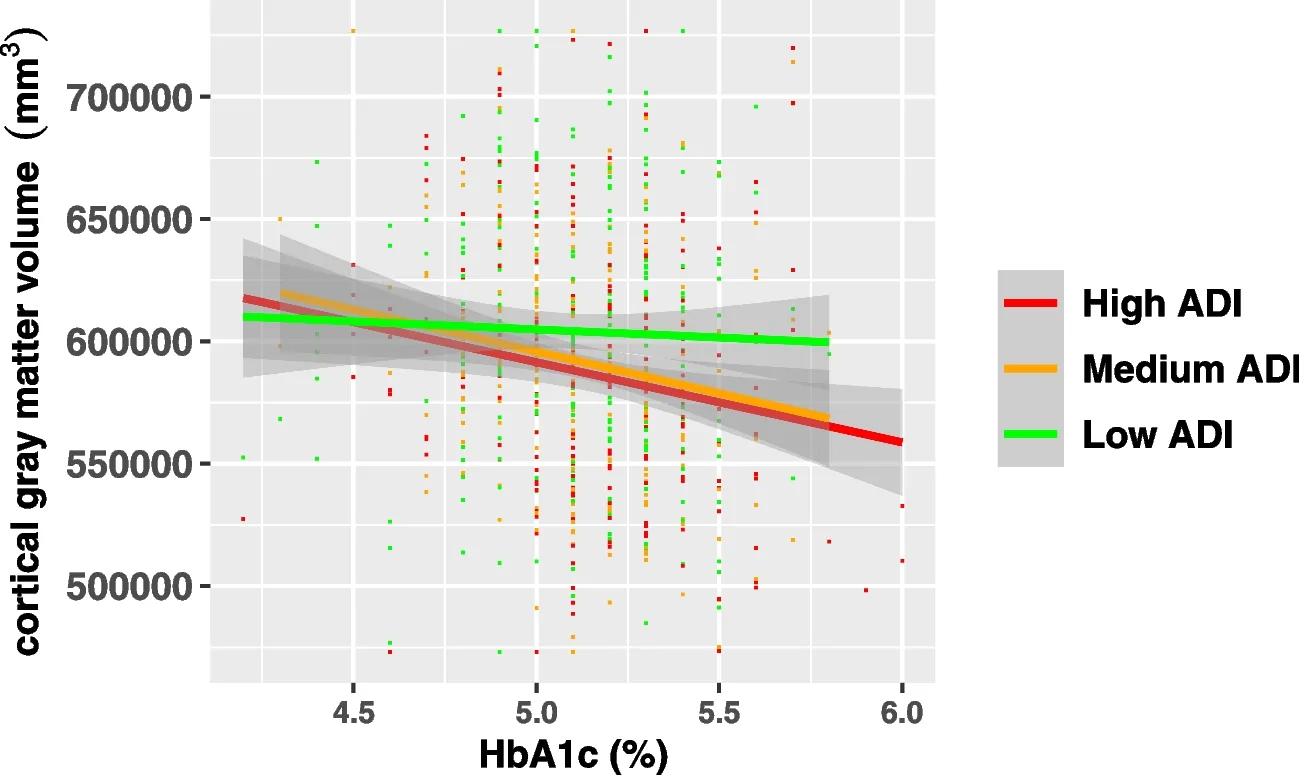
Relationships between HbA1c levels (%) and adjusted total cortical gray matter volume (GMV) in children from neighborhoods with low, medium, and high area deprivation index (ADI). Lines represent interaction model regression fits and shaded areas around the fitted lines represent standard error of the fits. This figure shows significant negative correlation between HbA1c total cortical GMV among residents of medium and high ADI neighborhoods

HbA1c by ADI interactions were marginally significant for global brain measurements [GMV: β (95% CI) = −0.029 (−0.062, 0.004), FDR corrected *P* = 0.087; mean CT: β (95% CI) = −0.062 (−0.128, 0.004)], FDR corrected *P* = 0.087].Additional descriptive analysis examined the associations between HbA1c and global brain measures within each ADI tertile. Higher HbA1c was associated with lower total GMV in the medium ADI group (β (95% CI) = −0.087 (−0.158, −0.015), FDR corrected* P* = 0.036), and the high ADI group (β (95% CI) = −0.071 (−0.133, −0.010), FDR corrected* P* = 0.024), but not the low ADI group (β (95% CI) = −0.004 (−0.067, 0.059), FDR corrected* P* = 0.897) (Fig. 2, Supplemental Table S4).

Higher HbA1c was associated with lower mean CT in the high ADI group (β (95% CI) = −0.156 (−0.278, −0.034), FDR corrected* P* = 0.024), but not the medium ADI (β (95% CI) = −0.121 (−0.264, 0.021), FDR corrected* P* = 0.095), nor low ADI group (β (95% CI) = 0.019 (−0.106, 0.144), FDR corrected* P* = 0.897) (Supplemental Table S4).

### Sensitivity analysis

As a sensitivity analysis, all primary models were additionally adjusted for BMI. The associations between HbA1c and total cortical GMV and mean CT remained significant, although the standardized coefficients were mildly attenuated (Supplemental Table S5). Additionally, associations between HbA1c and regional cortical GMV and CT remained statistically significant after adjustment for BMI, with little overall change in effect sizes (Supplemental Table S6-S7).

## Discussion

The aim of our study is twofold: first, to examine the relationships between glycemic control and brain structures in healthy developing youth without diabetes; second, to understand the role of neighborhood socioeconomic deprivation in moderating these relationships. We observed that higher HbA1c was associated with lower cortical thickness and total gray matter volume. Although the HbA1c × ADI interaction was not significant with ADI modeled continuously, analyses stratified by ADI tertile showed that higher HbA1c was associated with lower total cortical GMV in the medium- and high ADI groups, and with lower mean cortical thickness only in the high ADI group. No significant associations were found in the low ADI group. These results suggest that biological variation in glycemic control may only translate into measurable brain differences under conditions of socioeconomic adversity.

Our results are consistent with prior studies that demonstrated lower GMVs in youth with chronic exposure to high levels of glucose (Mauras et al., 2015; Mazaika et al., 2016; Perantie et al., 2007). Since our study included a sample of youth without diabetes, it presents novel findings in a population that has not been studied extensively, and our study results suggest that subtle dysregulation in glycemic control may be associated with structural brain differences detectable *before* the onset of diabetes. Our primary finding that higher HbA1c levels associated with smaller brain volumes supports previous research on the detrimental impact of poor glycemic control on brain regions such as the prefrontal cortex and limbic areas (Canário et al., 2024; Choi et al., 2020). The ROI analysis found that some of the effects are specific to particular cortices which is consistent with the notion that chronically elevated glucose levels can disrupt the normal development of brain regions responsible for executive functioning, emotional regulation, and cognitive processing (Coccaro et al., 2021; Garfield et al., 2021; Roy et al., 2020; Vincent & Hall, 2015; Zhang et al., 2022).

We observed that a 1% increase in HbA1c was linked to a 1.6% decrease in total cortical GMV and 0.9% decrease in mean CT, which could suggest an association between chronic exposure to elevated glucose and the brain. Multiple studies have shown that decreases in total cortical GMV and mean CT can have clinical implications in various neurological and psychiatric disorders, such as dementia, schizophrenia, and depression in adults (Li et al., 2020; Patel et al., 2021; Wennberg et al., 2016; Y. Zhang et al., 2016). A recent study by Shin et al. showed no significant relationship between HbA1c and mean CT in healthy adults, but a negative relationship between HbA1c and mean CT only in adults with prediabetes and diabetes (Shin et al., 2023). Our study challenges this finding, as there is a negative relationship between HbA1c and mean CT in healthy developing youth. This indicates that higher HbA1c may have a stronger association with cortical structure, as the adolescent brain is particularly sensitive to environmental influences.

Our study is also among the first to examine how neighborhood socioeconomic deprivation impacts the relationship between glycemic control and brain structure in youth without diabetes. Most prior work has focused on people with diabetes. In analyses stratified by ADI tertile, the negative association between HbA1c and cortical structure appeared stronger in more deprived neighborhoods. This pattern is consistent with evidence that neighborhood disadvantage is associated with differences in youth brain structure and neurodevelopment (Hackman et al., 2021; Rakesh et al., 2022). Socioeconomic context may amplify biological risk through several pathways, such as chronic stress, less access to health-promoting resources, and structural barriers to care. From this view, elevated HbA1c may not affect the developing brain equally everywhere; its impact may depend on neighborhood deprivation. Because the continuous interaction was not significant, these findings need confirmation in future studies.

Our study has several limitations. First, the within-tertile findings should be interpreted cautiously and treated as hypothesis-generating, because the HbA1c × ADI interaction was only marginally significant after FDR correction when ADI was modeled as a continuous variable. More studies are needed to see whether these patterns replicate and whether associations between glycemic control and cortical measures differ by level of neighborhood socioeconomic deprivation. Second, because our analyses are cross-sectional, we cannot draw causal conclusions about the relationships among glycemic control, neighborhood deprivation, and brain structure. HbA1c was available at only one timepoint in ABCD Release 4.0, which ruled out longitudinal analyses of glycemic control. Longitudinal studies will be needed to establish the order of these relationships and to test whether the effects of neighborhood deprivation on brain structure build up over time. Third, although we adjusted for a range of sociodemographic factors, residual confounding is possible. Associations remained significant after adjustment for BMI in sensitivity analyses. However, BMI is an imperfect measure of adiposity in youth, and other unmeasured factors could influence both glycemic control and brain structure, such as diet, physical activity, and sleep. Fourth, our analytic sample (N = 705) was a small subset of the ABCD cohort. Most exclusions happened because HbA1c was collected only before March 2020, and we used complete-case analysis. Included and excluded participants differed on some characteristics (Supplemental Table S1), which may introduce selection bias and limit how well the findings generalize to the full ABCD cohort and the broader U.S. youth population. Finally, our sample included only youth without diabetes, so the findings may not apply to youth with diabetes or other chronic metabolic disorders.

## Conclusions

In youth without diabetes, higher HbA1c was associated with lower cortical thickness and smaller global and regional cortical gray matter volumes. In analyses stratified by neighborhood deprivation, HbA1c was negatively associated with cortical gray matter volume in the medium- and high ADI groups, but not in the low ADI group. It was negatively associated with cortical thickness only in the high ADI group. These findings suggest that glycemic levels below the diabetes threshold are linked to differences in brain structure, and that this link may be stronger in more deprived neighborhoods. Early metabolic health deserves further study as a possible target for intervention to support brain development, especially among youth facing socioeconomic disadvantage.

## Supplementary Information

Below is the link to the electronic supplementary material.

Supplementary file1 (DOCX 4657 KB)

## Data Availability

Data used in the preparation of this article were obtained from the Adolescent Brain Cognitive Development (ABCD) Study (https://abcdstudy.org), held in the National Institute of Mental Health (NIMH) Data Archive (NDA). This is a multisite, longitudinal study designed to recruit more than 10,000 children aged 9 to 10 years and follow them over 10 years into early adulthood. The ABCD Study is supported by the NIH and additional federal partners under award numbers U01DA041048, U01DA050989, U01DA051016, U01DA041022, U01DA051018, U01DA051037, U01DA050987, U01DA041174, U01DA041106, U01DA041117, U01DA041028, U01DA041134, U01DA050988, U01DA051039, U01DA041156, U01DA041025, U01DA041120, U01DA051038, U01DA041148, U01DA041093, U01DA041089, U24DA041123, and U24DA041147. A full list of supporters is available at https://abcdstudy.org/federal-partners.html. A listing of participating sites and a complete listing of the study investigators can be found at https://abcdstudy.org/consortium_members/. ABCD Study consortium investigators designed and implemented the study and/or provided data but did not necessarily participate in analysis or writing of this report. This manuscript reflects the views of the authors and may not reflect the opinions or views of the NIH or ABCD Study consortium investigators. The ABCD Study data repository grows and changes over time. The ABCD data used in this report came from https://doi.org/10.15154/cqdy-5453. Additional support for this work was made possible from NIEHS R01-ES032295 and R01-ES031074. Code availability The R scripts used for the current analysis are available on reasonable request.

## Code availability

The R scripts used for the current analysis are available on reasonable request.
